# Supporting Sustainable Development of Water Resources: A Social Welfare Maximization Game Model

**DOI:** 10.3390/ijerph16162896

**Published:** 2019-08-13

**Authors:** Mingjing Guo, Ziyu Jiang, Yan Bu, Jinhua Cheng

**Affiliations:** 1 School of Economics and Management, China University of Geosciences, Wuhan 430074, China; 2 Research Center of Resource and Environmental Economics, China University of Geosciences, Wuhan 430074, China; 3 School of Economics and Management, Dalian University of Technology, Dalian 116024, China

**Keywords:** water pollution, maximization of social welfare, game, total sewage control

## Abstract

Water can carry a boat but can also overturn it (human societal sustainable development). Governments faced aquatic ecosystem restoration and preservation challenges following the establishment of the United Nations Sustainable Development Goals. This paper proposes a social welfare maximization game model to analyze the dominant strategy equilibrium of enterprise-1 and enterprise-2 based on welfare maximization under the total sewage emission control policy. Under the aforementioned control policy, a stricter total sewage emission control of an enterprise corresponds to a lower enterprise output and a higher output of a competing enterprise; that is, the profit transfer effect occurs. When the government implements a relatively strict total sewage emission control policy for an enterprise, it is beneficial to reduce the amount of sewage emission from an enterprise but has no impact on the amount of sewage emission from a competing enterprise; however, the amount of sewage reduction of both enterprises will increase. If the government does not provide capital and technical support to enterprise-2, then enterprise-1 and enterprise-2 should implement total quantity control policies with different rigor degrees to avoid the one-size-fits-all phenomenon. To maximize social welfare, the government should adjust the total sewage emission control policy in time according to sewage stock changes and focus more on enterprises with insufficient capital and poor technical skills and provide financial and technical support.

## 1. Introduction

Water is an important natural resource to maintain the basic survival of humans, production and societal and economic development. With the accelerated pace of industrialization and increased urbanization, water resource security is threatened, and water resource crises emerge [1]. According to the United Nations World Water Development Report 2018, Nature-based Solutions for Water, the global demand for water resources is increasing by 1% per year. Currently, approximately 3.6 billion people live in water-deficient areas, and the number of people experiencing water deficiencies may increase from 4.8 billion to 5.7 billion by 2050 [2]. At the same time, the increasingly serious problem of water pollution further aggravates the conflict between water supply and demand [3]. The main causes of water pollution are industrial pollution, anthropogenic activities, and agricultural pollution [4,5,6,7], which cause deterioration of the ecological environment [8] and human health problems [9,10] and have varying degrees of impact on the socioeconomic development [3] and social well-being of various countries [4]. Water can carry a boat but can also overturn it (sustainable development of human society). Governments face the challenge of restoring and preserving aquatic ecosystems in accordance with the United Nations Sustainable Development Goals (SDG 6) [11].

In the European Union (EU), water pollution, over-abstraction, and hydro-morphological alterations have been implicated as the major significant pressures for European water bodies [12]. Currently, more than 700 emerging pollutants, their metabolites and transformation products are listed as present in the European aquatic environment. Emerging pollutants from urban or industrial wastewater treatment plants are directly discharged into rivers where their environmental fate is of concern (degradation, sorption at the sediment, and transport in the aqueous phase) [13]. The EU Water Framework Directive 2000/60/EC (WFD) aimed to introduce a new era for European water management. However, fifteen years after the WFD was introduced, achieving its objectives remains challenging, with 47% of EU surface waters failing to reach good ecological status in 2015—a central objective of the EU water legislation [14]. According to the Commission, in 2011, approximately 143 towns did not have suitable sewage systems, creating hazards for the environment and public health. Consequently, Italy is under infringement proceedings by the EU in accordance with EU Directive 271/91. The Italian sewage systems are still currently experiencing many failures and do not meet the EU requirements; many urban agglomerations over 10.000 inhabitants discharge sewage without any proper treatment [15].

More than 70% of people who lack sanitation, or 1.8 billion people, live in Asia [13]. In 2013, the Ministry of Environment in Korea reported that more than one hundred leading Korean enterprises, including Samsung, Hyundai, SK, and LG, had been discharging wastewater illegally, worsening the situation [16]. In China, which has a rapidly growing economy, water is a scarce resource as only 8% of the world’s freshwater is available to meet the needs of 22% of the world’s population. However, 33% of industrial wastewater and 70% of household sewage are untreated and directly released into rivers and lakes. Moreover, 80% of China’s cities have no sewage treatment facilities, and water supplies in 90% of the cites are contaminated. Environmental degradation costs China nearly 9% of its annual gross domestic product [13]. Dong et al. evaluated the relative sustainability of the water infrastructure of 157 cities in China and found only 69 cities with high sustainability [17]. The total sewage emission in Beijing, Shanghai, and Guangzhou, China’s three most developed cities, shows an upward trend [18].

As early as the Water Pollution Prevention and Control Law of the People’s Republic of China of 2008, those who discharge water pollutants in excess of national or local emission standards for water pollutants or in excess of total emission control targets for key water pollutants are required to pay a fine of no less than two-times but no more than five-times the emission fee [19]. Although the government has established strict laws and regulations to restrict the emission of sewage, water pollution incidents still occur. One specific example is the city of Wuhan, the largest metropolis in central China, where 55% of its 108 lakes have been polluted to varying degrees according to the Wuhan Municipal Water Authority. As another example of a pollution accident, on 23 April 2014, two major water suppliers (Baihezui and Yushidun waterworks) in Wuhan city halted production at approximately 4 p.m. and 7 p.m., respectively, because the water in the Wuhan section of the Han River contained excessive amounts of ammonia and nitrogen, which exceeded the national standards. The water supply suspension caused a water shortage and affected 300,000 people [20]. First, the government sets the regulation that enterprises will be fined if their sewage emission exceeds the standard. Although the government can restrain the sewage emission of some enterprises, other enterprises are willing to accept the penalty and discharge excessive sewage to pursue efficiency. Second, excessive emission of enterprise sewage will directly affect the health of the residents living near the enterprise in the short term, which will lead to complex social problems. Finally, excess sewage does not easily degrade in the natural environment, and in the long term, the excess sewage will affect the sustainable development of the whole society with increasing sewage emission. Therefore, we focus on the problem of selecting a more reasonable strategy to ensure societal and economic development and reasonably control the emission of sewage.

Academic research on sustainable development of water resources covers a wide range, such as integrated water resource management [21,22], water resource carrying capacity [23,24,25], water resource efficiency [15,26,27], water quality management [28,29,30], and water resource allocation strategy [31], etc. The optimal allocation of water resources could be determined by the optimization method [32]. A series of optimization methods have been used to solve the allocation of water resources [33]. These techniques are useful to recognize the best management strategy to achieve a given set of objectives under different constraints [34]. The optimization methods include linear programming (LP) [35], nonlinear programming (NLP) [36], dynamic programming (DP) [37], and game model. Although game theory requires further development in the field of water resource management and other related fields, game theory is a well-known method used to reflect various important behaviors of the involved parties [38]. At present, most scholars mainly study water resource allocation based on a game model. For example, Kicsiny et al. adopted a discrete solution differential game model to distribute available water resources with maximum efficiency among different consumers [39]. Zanjanian et al. used the Graph Model for Conflict Resolution to resolve the nonquantitative conflict of water rights among Ilam’s dam organizational stakeholders [40]. Chhipi-Shrestha et al. proposed a multi-criteria, multi-decision-maker framework combining multi-criteria decision analysis (MCDA) and game theory for the selection of a sustainable water reuse application by multiple stakeholders [41]. Han et al. established a multi-agent game theory optimization model that could realize the maximization of common interests and individual interests [42]. Davijani et al. proposed a two-objective socioeconomic model (aimed at job creation) to determine the optimum allocation of water resources to industry, agriculture, and municipal water sectors [43]. Based on game theory, extensive research results have been obtained for water resource allocation, thus providing a sufficient decision-making basis for the rational allocation of water resources. Such results also provide the basis for the model hypothesis in Section 2 of this paper (this paper assumes that under the existing water resource allocation theory based on game theory and under the control of the government, enterprise-1 and enterprise-2 could obtain fair, sufficient, affordable, and continuous water resources supplies). The core research content of this paper is water pollution control.

However, only a few studies on water pollution control based on game models are available. Zeng et al. proposed a hybrid game theory and mathematical programming model (HGT-MPM) to solve trans-boundary water conflicts in the Guanting reservoir basin (GRB) using Zhangjiakou and Beijing as examples. To optimize water use and pollutant emission in the two cities, the net aggregate benefits from these activities were maximized, and the costs for water supply and pollution removal were reduced; thus, a water allocation model based on both water quality and quantity was developed [44]. Magnuszewski et al. used an observation protocol to collect data on the quality of relational practices, compared these data with the quantitative outcomes of game participants, and introduced a game-based approach to explore the effects of relational practices on the effectiveness of water governance [45].

Sustainable development of water resources is a serious and complicated problem. Based on the game model, sustainable development of water resources could be studied from two aspects: before water resource use (water resource allocation) and after water resource use (water pollution control). To further expand the application of game theory in water resource management and provide effective strategies for water pollution control, this paper implements Yanase’s research concept of using a differential game model to analyze the perfect competition between two countries in a third country’s market under the two environmental regulation policies of carbon tax and total control [46]. This paper proposes a social welfare maximization game model, sets strict sewage emission constraints, and studies how two enterprises (enterprise-1 and enterprise-2) produce products of the same quality and compete for sales in the same market. In addition, the question of how the government can formulate an effective sewage emission strategy is examined. Finally, the Markov-perfect Nash equilibrium results in a total sewage emission control policy that may maximize social welfare.

The remainder of this article is organized as follows. The second Section discusses the hypothesis of the model. Section 3.1 presents a static game analysis of complete information, Section 3.2 presents a dynamic differential game analysis, Section 3.3 presents a social welfare effect analysis, and Section 3.4 compares sewage stocks. Finally, Section 4 summarizes the paper and proposes suggestions to support the sustainable development of water resources.

## 2. Materials and Methods

Suppose that only two enterprises (enterprise-1 and enterprise-2) exist in a certain region of China. Under the control of the government, enterprise-1 and enterprise-2 could obtain fair, sufficient, affordable, and continuous water resource supplies. Enterprise-1 and enterprise-2 produce completely homogeneous products, and all products are sold in market-3. Enterprise-1 and enterprise-2 compete perfectly in market-3. The inverse demand function is $p=a-x_{i}-x_{j},\;a>0,\;i,\;j=1,\;2,\;\text{and}\;i\neq j$, where $\;x_{i}$ and $x_{j}$ are the number of goods produced by enterprise-1 and enterprise-2, respectively. The marginal cost of the products produced by the two enterprises does not change and is constant. For ease of calculation, assume that the marginal cost is zero. Then, the revenue of the enterprise could be expressed as $R_{i}=px_{i}=\left(a-x_{i}-x_{j}\right)x_{i}$. All symbols in this paper are explained in Appendix A (in Table A1).

The production process is accompanied by sewage emission, which causes damage to the regional water resource environment. With the accumulation of sewage, the sustainable development of regional water resources will be affected, and serious losses will occur. To control sewage emissions, suppose the local government implements water pollution control policies to reduce the water pollution. Assuming that the enterprise output per unit of product accompanied by ε units of sewage emission, the amount of sewage emission from enterprise *i* is $E_{i}=\varepsilon x_{i}-\alpha_{i},\;i=1,\;2$, where $\alpha_{i}$ is the amount of sewage reduction from enterprise *i.* (α  could be thought of as the amount of sewage purified by sewage treatment equipment). For ease of calculation, assume that ε = 1. Enterprise *i*’s emission reduction $\alpha_{i}$ units cost is $K_{i}\left(\alpha_{i}\right)=\frac{k_{i}\alpha_{i}^{2}}{2}$, where $k_{i}$ is the sewage emission reduction coefficient, which is used to measure the sewage emission reduction efficiency of enterprise *i*. A higher value of $k_{i}$ corresponds to lower emission reduction efficiency and thus a higher sewage emission reduction cost.

Suppose that the government sets a strict cap on the total sewage emission, $x_{i}-\alpha_{i}\leq \theta_{i}$, where $\theta_{i}$ represents the total sewage emission control set by the government for enterprise *i*. A lower value of $\;\;\theta_{i}$ corresponds to a stricter total sewage emission control policy for enterprise *i*. The consequences for violating the government’s strict cap on sewage emissions are similar to those for driving a motor vehicle while drunk in China, which can result in license suspension and a lifetime ban on operating vehicles [47]. Enterprises are shut down if they exceed the sewage emission standards set by the government. This policy differs from those in previous studies in which the government allows enterprises to discharge excess sewage as long as the enterprises pay a penalty fee for the excess sewage [44,48]. Therefore, we assume that both enterprise-1 and enterprise-2 could strictly comply with the total sewage emission control policy formulated by the government.

Suppose that enterprise-1 has sufficient funds, advanced technology, and relatively mature sewage emission reduction technology. Enterprise-2 lacks funds and technology, and sewage emission reduction has a considerable impact on the enterprise economy. Therefore, in a broad sense, the sewage emission reduction cost of enterprise-2 is higher than that of enterprise-1. To simplify the calculations, assume that $k_{1}=1\;\text{and}\;k_{2}=k>1$.

Suppose that the sewage emission stock in this area is S(t)  at time t, then:

(1) $$\dot{S}\left(t\right)=\frac{dS\left(t\right)}{dt}\left(\underset{i=1,\;2}{\sum }(x_{i}-\alpha_{i})-\beta \right)-\delta S\left(t\right),\;\;S\left(0\right)=S_{0}>0$$

In the equation, δ>0, where δ represents the natural degradation rate of sewage, and $\beta =\zeta \left(E_{1}+E_{2}\right),\;\mathrm{where}\;\zeta \geq 0$, β  represents the amount of sewage treatment from government, and ζ represents the government’s sewage emission reduction coefficient; a larger  ζ corresponds to a greater intensity of sewage treatment by the government.

**Figure 1:**
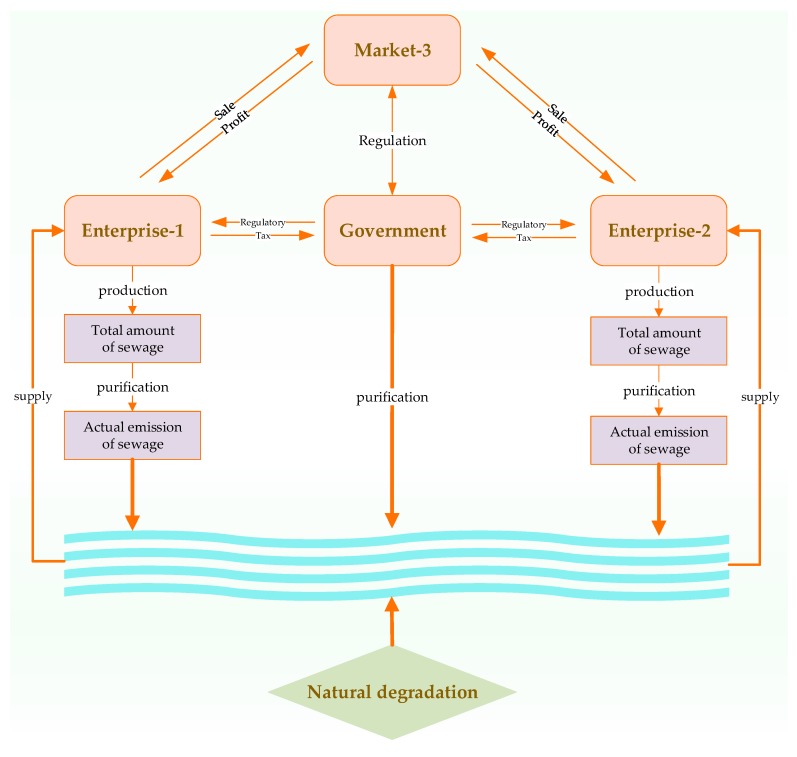
Schematic diagram of the whole game process.

Finally, we assume that the environmental damage function of sewage stock to the water resource environment is $D\left(S\right)=S^{2}$. Figure 1 shows a schematic diagram of the whole game process.

## 3. Results and Discussion

Assume that enterprise-1 and enterprise-2 only consider the profit maximization when manufacturing regardless of changes in the water resource environment. Government decision-making considers both economic growth and environmental protection.

### 3.1. Static Games of Complete Information

According to the model assumptions in Section 2, the profit function of enterprise *i* is:

(2) $$\pi_{i}=\left(1-\rho \right)R_{i}-K_{i}\left(\alpha_{i}\right)-\mu \left(x_{i}-\alpha_{i}\right)=\left(1-\rho \right)\left(a-x_{i}-x_{j}\;\right)x_{i}-\frac{k_{i}\alpha_{i}^{2}}{2}-\mu \left(x_{i}-\alpha_{i}\right)$$

In Equation (2), $\left(1-\rho \right)R_{i}$ represents the gross profit after enterprise *i* pays taxes, $K_{i}\left(\alpha_{i}\right)$ represents the cost of reducing sewage discharged by $\alpha_{i}$ units, and $\mu \left(x_{i}-\alpha_{i}\right)$ represents the cost paid by enterprise *i* to the government for discharging $x_{i}-\alpha_{i}$ units of sewage.

Moreover, enterprises should strictly comply with the sewage emission ceiling $\theta_{i}$ set by the government, that is, $x_{i}-\alpha_{i}\leq \theta_{i}$; therefore, we construct the following Lagrange function:

(3) $$L_{i}=\left(1-\rho \right)\left(a-x_{i}-x_{j}\;\right)x_{i}-\frac{k_{i}\alpha_{i}^{2}}{2}-\mu \left(x_{i}-\alpha_{i}\right)+\lambda_{i}\left(\theta_{i}-x_{i}+\alpha_{i}\right)$$

Substituting $\;k_{1}=1$ and $k_{2}=k>1$ into Equation (2) yields the following equation:

(4) $$\{\begin{array}{c} L_{1}=\left(1-\rho \right)\left(a-x_{1}-x_{2}\;\right)x_{1}-\frac{\alpha_{1}^{2}}{2}-\mu \left(x_{1}-\alpha_{1}\right)+\lambda_{1}\left(\theta_{1}-x_{1}+\alpha_{1}\right)\\ L_{2}=\left(1-\rho \right)\left(a-x_{2}-x_{1}\;\right)x_{2}-\frac{k\alpha_{2}^{2}}{2}-\mu \left(x_{2}-\alpha_{2}\right)+\lambda_{2}\left(\theta_{2}-x_{2}+\alpha_{2}\right) \end{array}$$

In Equation (4), ρ is the tax rate collected by the government, and μ is the fee paid by the enterprise to the government for per unit sewage emission. For ease of calculation, we set Equations (10a) and (10b) are substituted into Equation (9) to obtain the differential equation o ρ=0.2 and μ=1.

To maximize profits, each enterprise chooses output ${\tilde{x}}_{i}\;$ and the amount of sewage reduction ${\tilde{\alpha }}_{i}\;$ under the optimal path, and the Cournot–Nash equilibrium could be obtained as follows:

(5a) $$\{\begin{array}{c} {\tilde{x}}_{1}=\frac{a\left(20k+16\right)+\theta_{1}\left(25k+40\right)-20k\theta_{2}}{65k+88}\\ {\tilde{\alpha }}_{1}=\frac{a\left(20k+16\right)-\left(40k+48\right)\theta_{1}-20k\theta_{2}}{65k+88} \end{array}$$

(5b) $$\{\begin{array}{c} {\tilde{x}}_{2}=\frac{36a-20\theta_{1}+65k\theta_{2}}{65k+88}\\ {\tilde{\alpha }}_{2}=\frac{36a-20\theta_{1}-88\theta_{2}}{65k+88} \end{array}$$

Output ${\tilde{x}}_{i}$ and the amount of sewage reduction ${\tilde{\alpha }}_{i}$ under the equilibrium condition are related to the total sewage emission control $\theta_{1}$ and $\theta_{2}$ in the two regions. From the Nash equilibrium results obtained above, the following conclusions can be drawn:

(1) $\frac{\partial {\tilde{x}}_{1}}{\partial \theta_{1}}=\frac{25k+40}{65k+88}>0$, that is, ${\tilde{x}}_{1}$ is an increasing function of $\;\theta_{1}$. When the total sewage emission control of enterprise-1 tends to be strict, that is, the value of $\theta_{1}$ decreases, the output ${\tilde{x}}_{1}$ of enterprise-1 decreases. $\frac{\partial {\tilde{x}}_{1}}{\partial \theta_{2}}=-\frac{20k}{65k+88}<0$, that is, ${\tilde{x}}_{1}$ is a decreasing function of $\theta_{2}$. When the total sewage emission control of enterprise-2 tends to be strict, that is, the value of $\theta_{2}$ decreases, the output ${\tilde{x}}_{1}$ of enterprise-1 correspondingly increases.

$\frac{\partial {\tilde{x}}_{2}}{\partial \theta_{2}}=\frac{65k}{65k+88}>0$, that is, ${\tilde{x}}_{2}$ is an increasing function of $\;\theta_{2}$. When the total sewage emission control of enterprise-2 tends to be strict, that is, the value of $\theta_{2}$ decreases, the output ${\tilde{x}}_{2}$ of enterprise-2 decreases. $\frac{\partial {\tilde{x}}_{2}}{\partial \theta_{1}}=-\frac{20}{65k+88}<0$, that is, ${\tilde{x}}_{2}$ is a decreasing function of $\theta_{1}$. When the total sewage emission control of enterprise-1 tends to be strict, that is, the value of $\theta_{1}$ decreases, the output ${\tilde{x}}_{2}$ of enterprise-2 correspondingly increases, i.e., the profit transfer effect occurs.

(2) $\frac{\partial {\tilde{\alpha }}_{1}}{\partial \theta_{1}}=-\frac{40k+48}{65k+88}<0$, that is, ${\tilde{\alpha }}_{1}$ is a decreasing function of $\theta_{1}$. When the total sewage emission control of enterprise-1 tends to be strict, that is, the value of $\theta_{1}$ decreases, the amount of sewage reduction ${\tilde{\alpha }}_{1}$ of enterprise-1 increases. $\frac{\partial {\tilde{\alpha }}_{2}}{\partial \theta_{1}}=-\frac{20}{65k+88}<0$, that is, ${\tilde{\alpha }}_{2}$ is a decreasing function of $\theta_{1}$. When the total sewage emission control of enterprise-1 tends to be strict, that is, the value of $\theta_{1}$ decreases, the amount of sewage reduction ${\tilde{\alpha }}_{2}$ of enterprise-2 increases.

Similarly, when the total sewage emission control of enterprise-2 tends to be strict, that is, the value of $\theta_{2}$ decreases, the amount of sewage reduction ${\tilde{\alpha }}_{1}\;$ and ${\tilde{\alpha }}_{2}$ of both enterprise-1 and enterprise-2 increases, respectively. In addition, ${\tilde{E}}_{1}={\tilde{x}}_{1}-{\tilde{\alpha }}_{1}=\theta_{1}\;\mathrm{and}\;{\tilde{E}}_{2}={\tilde{x}}_{2}-{\tilde{\alpha }}_{2}=\theta_{2}$. According to $\frac{\partial {\tilde{E}}_{1}}{\partial \theta_{1}}=1>0,\;\frac{\partial {\tilde{E}}_{1}}{\partial \theta_{2}}=0$, when the total sewage emission control of enterprise-1 tends to be strict, the amount of sewage reduction of enterprise-1 is reduced, while the amount of sewage reduction of enterprise-2 is not affected. Similarly, $\frac{\partial {\tilde{E}}_{2}}{\partial \theta_{2}}=1>0,\;\frac{\partial {\tilde{E}}_{2}}{\partial \theta_{1}}=0;\;$ thus, when the total sewage emission control of enterprise-2 tends to be strict, the amount of sewage reduction of enterprise-2 is reduced, while the amount of sewage reduction of enterprise-1 is not affected.

(3) Assume that the two regions have the same strict total sewage control policy, that is, $\;\theta_{1}=\theta_{2}=\theta .\;$ From the above equation, one can conclude that ${\tilde{x}}_{1}-{\tilde{x}}_{2}=\frac{20\left(a-3\theta \right)\left(k-1\right)}{65k+88}>0$ (because ${\tilde{\alpha }}_{2}>0$; thus, a−3θ>0); that is, the equilibrium output level of enterprise-1 is higher than that of enterprise-2. At the same time, ${\tilde{\pi }}_{1}>{\tilde{\pi }}_{2}$, and the equilibrium profit level of enterprise-1 is higher than that of enterprise-2.

### 3.2. Dynamic Differential Game

Assume that under the total sewage emission control policy of the two enterprises, the Markov-perfect Nash equilibrium is $\left({\tilde{\theta }}_{i}\left(S\right),{\tilde{\theta }}_{j}\left(S\right)\right),i,\;j=1,\;2,\;i\neq j,$ and ${\tilde{\theta }}_{i}\left(S\right)$ is the total sewage emission control policy formulated by the government for enterprise i to maximize the social welfare under the condition that ${\tilde{\theta }}_{j}\left(S\right)$ is determined. Since the water environment quality is affected by variable S and S changes with time  t, the welfare of enterprise i is also a function of time t, which can be set as:

(6) $$W_{i}=\left(1-\rho \right)\left(a-x_{i}\left(\theta_{i},\theta_{j}\right)-x_{j}\left(\theta_{i},\theta_{j}\right)\right)x_{i}\left(\theta_{i},\theta_{j}\right)-\frac{k_{i}\alpha_{i}^{2}}{2}-\mu \left(x_{i}\left(\theta_{i},\theta_{j}\right)-\alpha_{i}\right)$$

The performance function of the government is as follows:

(7) $$G=\rho \left[a-x_{i}\left(\theta_{i},\theta_{j}\right)-x_{j}\left(\theta_{i},\theta_{j}\right)\right]\left(x_{i}\left(\theta_{i},\theta_{j}\right)+x_{j}\left(\theta_{i},\theta_{j}\right)\right)+\mu \left(x_{i}\left(\theta_{i},\theta_{j}\right)-\alpha_{i}+x_{j}\left(\theta_{i},\theta_{j}\right)-\alpha_{j}\right)\;-\frac{c{\left(\zeta \left(x_{i}\left(\theta_{i},\theta_{j}\right)-\alpha_{i}+x_{j}\left(\theta_{i},\theta_{j}\right)-\alpha_{j}\right)\right)}^{2}}{2}$$

We believe that the enterprises and the ecological environment constitute a community of common destiny. The maximization of social welfare indicates that the government and enterprises work together to ensure the sustainable development of both the social economy and the ecological environment and ultimately achieve sustainable development of the region overall. Therefore, the objective function is the maximization of social welfare, namely:

(8) $$W=\underset{\theta_{i},\theta_{j}}{\max}\int_{0}^{\infty }\left(W_{1}+W_{2}+G\right)e^{-rt}dt$$

where r>0 is the discount rate, and the constraint condition of the objective function is Equation (1).

We define V(S) as a value function and substitute Equations (6) and (7) into Equation (8). Through transformation, we deduce that $\tilde{V}\left(S\right)$ satisfies the following Hamilton–Jacobi–Bellman equation:

(9) $$\begin{array}{cc} r\tilde{V}(S)\;=\;\underset{\theta_{i},\theta_{j}}{\max} & \{\left[a-x_{i}\left(\theta_{i},\theta_{j}\right)-x_{j}\left(\theta_{i},\theta_{j}\right)\right]\left(x_{i}\left(\theta_{i},\theta_{j}\right)+x_{j}\left(\theta_{i},\theta_{j}\right)\right)-\frac{\alpha_{i}^{2}}{2}-\frac{k_{j}\alpha_{j}^{2}}{2}\\ & -\frac{c{\left(\zeta \left(x_{i}\left(\theta_{i},\theta_{j}\right)-\alpha_{i}+x_{j}\left(\theta_{i},\theta_{j}\right)-\alpha_{j}\right)\right)}^{2}}{2}-S^{2}\\ & +{\tilde{V}}^{\prime }\left(S\right)\left[\left(1-\zeta \right)\left(x_{i}\left(\theta_{i},\theta_{j}\right)-\alpha_{i}+x_{j}\left(\theta_{i},\theta_{j}\right)-\alpha_{j}\right)-\delta S\right]\} \end{array}$$

where $i,\;j=1,\;2,\;i\neq j,\;\frac{c{\left(\zeta \left(x_{i}\left(\theta_{i},\theta_{j}\right)-\alpha_{i}+x_{j}\left(\theta_{i},\theta_{j}\right)-\alpha_{j}\right)\right)}^{2}}{2}$ is the government’s sewage emission reduction expense, and ζ is the government’s sewage emission reduction coefficient. Suppose that the government should achieve at least a 20% sewage emission reduction. For ease of calculation, we set $\zeta =0.2\;\mathrm{and}\;\frac{c\zeta^{2}}{2}=1$. Moreover, in a Markov-perfect Nash equilibrium, for any S>0, the value function must satisfy $\underset{t\to \infty }{\lim}\tilde{V}\left(S\right)e^{-rt}=0$.

By substituting Equations (5a) and (5b) into Equation (9), the following solutions could be obtained:

(10a) $${\tilde{\theta }}_{1}=\frac{a\left(375k^{2}+125k+560\right)+\left(1400k^{2}+3224k\right){\tilde{V}}^{\prime }\left(S\right)}{2500k^{2}+10060k+3880}$$

(10b) $${\tilde{\theta }}_{2}=\frac{a\left(125k^{2}+1495k-560\right)+\left(3104+1720k-200k^{2}\right){\tilde{V}}^{\prime }\left(S\right)}{2500k^{2}+10060k+3880}$$

Equations (10a) and (10b) are substituted into Equation (9) to obtain the differential equation of $\tilde{V}\left(S\right)$. Since the objective function is a quadratic function, the linear Markov strategy shows that the value function is also a quadratic function of S. Assuming that the value function form is $\tilde{V}\left(S\right)=AS^{2}/2+BS+C$, then ${\tilde{V}}^{\prime }\left(S\right)=AS+B$, and by substituting the latter equation into Equations (10a) and (10b), we can obtain:

(11a) $${\tilde{\theta }}_{1}=\frac{a\left(375k^{2}+125k+560\right)+\left(1400k^{2}+3224k\right)\left(AS+B\right)}{2500k^{2}+10060k+3880}$$

(11b) $${\tilde{\theta }}_{2}=\frac{a\left(125k^{2}+1495k-560\right)+\left(3104+1720k-200k^{2}\right)\left(AS+B\right)}{2500k^{2}+10060k+3880}$$

By substituting Equations (11a), (11b), (5a) and (5b) into Equation (9), when the government uses a linear strategy, unknown variables A, B, and C must satisfy:

(12) $$\{\begin{array}{c} \phi_{2}A^{2}-\left(\frac{r}{2}+\delta \right)A-1=0\;\\ \left(2\phi_{2}A-\delta -r\right)B+\phi_{1}A=0\\ \left(\phi_{2}B+\phi_{1}\right)B+\phi_{3}-rC=0 \end{array}.\;$$

where:

$$\phi_{1}=\frac{ak\left(1000k+3240\right)}{6250k^{2}+25150k+9700},\phi_{2}=\frac{1200k^{2}+4944k+3104}{6250k^{2}+25150k+9700},\phi_{3}=\frac{a^{2}\left(1250k^{2}+4975k+2225\right)}{6250k^{2}+25150k+9700}$$

Equation (11) could be solved as follows:

(13) $$\{\begin{array}{l} A=\frac{r+2\delta \pm \sqrt{{\left(r+2\delta \right)}^{2}+16\phi_{2}}}{4\phi_{2}}\\ B=\frac{\phi_{1}A}{r+\delta -2\phi_{2}A}\\ C=\frac{\phi_{3}+\left(\phi_{1}+\phi_{2}B\right)B}{r} \end{array}$$

Notably, A>0 does not meet the conditions and must be discarded. The detailed proof process is reported in the literature [46]. Therefore, $A=\frac{r+2\delta -\sqrt{{\left(r+2\delta \right)}^{2}+16\phi_{2}}}{4\phi_{2}}<0\;\mathrm{and}\;B<0$, and we can obtain $\frac{\partial {\tilde{\theta }}_{1}}{\partial S}=\frac{1400k^{2}+3224k}{2500k^{2}+10060k+3880}A<0$ and $\frac{\partial {\tilde{\theta }}_{2}}{\partial S}=\frac{3104+1720k-200k^{2}}{2500k^{2}+10060k+3880}A<0\;$(notably, we did not provide the upper limit of k previously, but when k→∞, enterprise-1 and enterprise-2 lose the meaning of the game; thus, we assume that 1<k<10). Therefore, one can conclude that a larger sewage emission stock in water resources corresponds to a stricter total sewage emission control policy requirement, and a smaller sewage emission stock in water resources corresponds to a less strict total sewage emission control policy requirement.

### 3.3. Analysis of the Social Welfare Effect

In the case that the government needs to achieve a 20% sewage emission reduction and the government unit sewage emission reduction cost c=50, only the value of k will affect the social welfare function. The two cases of k=1 and k>1 are compared. In the equilibrium case, the social welfare function is $V\left(S\right)=AS^{2}/2+BS+C$, and we, therefore, only need to compare the sizes of  A,B, and C. When  k=1, A,B, and C are represented as $A_{1},B_{1},\;\mathrm{and}\;C_{1}$, respectively. When k>1, A, B, and C are represented as $A_{k},B_{k},\;\mathrm{and}\;C_{k}$, respectively.

About A: Since $\phi_{2}>0$, $\frac{1}{\phi_{2}}$ is an increasing function of  k. In $A=\frac{r+2\delta -\sqrt{{\left(r+2\delta \right)}^{2}+16\phi_{2}}}{4\phi_{2}}$,$\;r+2\delta -\sqrt{{\left(r+2\delta \right)}^{2}+16\phi_{2}}<0,$ and A is therefore a decreasing function of k, and $\;A_{k}<A_{1}<0$.

About B: Since $B=\frac{\phi_{1}A}{r+\delta -2\phi_{2}A},\;A<0$, and, therefore, B<0. When k>1, the proof reported in the literature [46] shows that $B_{k}<B_{1}<0$.

**Figure 2:**
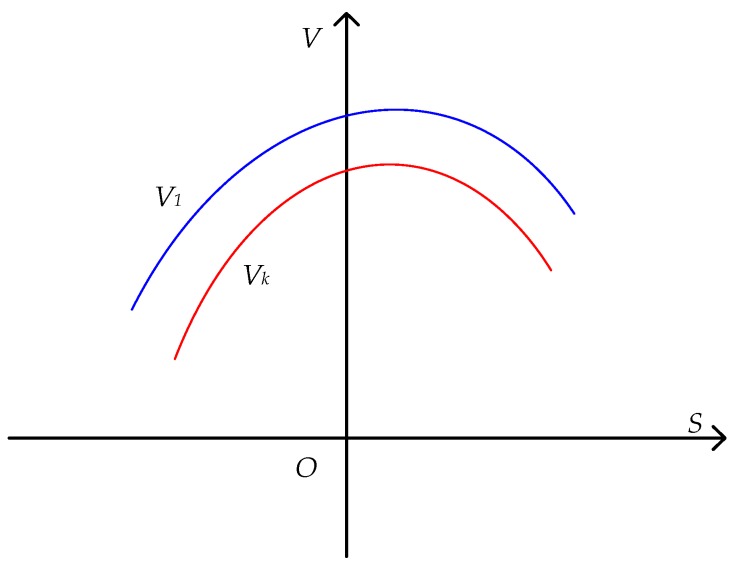
Comparison of social welfare function V.

About C: Since $rC=\phi_{2}{\left[B+\frac{\phi_{1}}{2\phi_{2}}\right]}^{2}+\phi_{3}-\frac{\phi_{1}{}^{2}}{4\phi_{2}}$ and $\phi_{3}-\frac{\phi_{1}{}^{2}}{4\phi_{2}}>0$, C>0. The proof in the literature [46] shows that $\;0<C_{k}<C_{1}.$ The function images of the social welfare functions $V_{1}\left(S\right)$ and $V_{k}\left(S\right)$ are shown in Figure 2. Under the total sewage emission control policy, for a given quantity of sewage stock  S, reducing the sewage emission reduction cost of enterprise-2 is conducive to increasing the regional welfare.

### 3.4. Comparison of the Sewage Stock

According to Equation (13), the sewage emission stock at time t in the region is:

(14) $$\begin{array}{c} E\left(S\right)=0.8\left({\tilde{\theta }}_{1}-{\tilde{\theta }}_{2}\right)-\delta S=\frac{1000ak^{2}+3240ak+\left(2400k^{2}+9888k+6208\right)\left(AS+B\right)}{6250k^{2}+25150k+9700}-\delta S\\ \;=\frac{1000ak^{2}+3240ak+\left(2400k^{2}+9888k+6208\right)B}{6250k^{2}+25150k+9700}\\ \;-\left[\frac{\left(2400k^{2}+9888k+6208\right)A}{6250k^{2}+25150k+9700}-\delta \right]S \end{array}$$

The sewage emission stocks under the two cases of k=1 and k>1 are compared, and E(S) is expressed as $E_{1}\left(S\right)$ and $E_{k}\left(S\right)$, respectively. Then:

(15) $$\begin{array}{cc} {E^{\prime }}_{k}\left(S\right)-{E^{\prime }}_{1}\left(S\right) & =\frac{\left(2400k^{2}+9888k+6208\right)A_{k}}{6250k^{2}+25150k+9700}-\frac{9248}{20500}A_{1}\\ & =\frac{\sqrt{{\left(r+2\delta \right)}^{2}+16\phi^{1}{}_{2}}-\sqrt{{\left(r+2\delta \right)}^{2}+16\phi^{k}{}_{2}}}{2} \end{array}$$

Since $\phi_{2}>0$, $\phi_{2}\;$ is a decreasing function of k, and therefore $\phi^{1}{}_{2}>\phi^{k}{}_{2}>0$ and $0<{E^{\prime }}_{1}\left(S\right)<{E^{\prime }}_{k}\left(S\right)$; the straight line of $E_{1}\left(S\right)$ is steeper than the straight line of $E_{k}\left(S\right)$. Through further analysis, $0<E_{1}\left(0\right)<E_{k}\left(0\right)\;$ (see Appendix B for the proof), and the graph of $E_{k}\left(S\right)$ is, therefore, above the graph of $E_{1}\left(S\right).$

**Figure 3:**
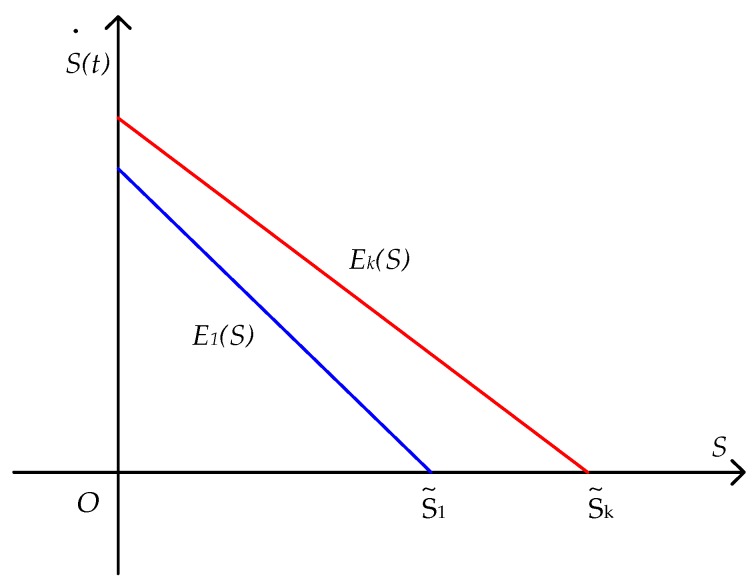
Comparison of the sewage stocks.

The function image of the sewage stock is shown in Figure 3. Reducing the sewage emission reduction cost of enterprise-2 is conducive to reducing the sewage stock in this region, and the steady-state sewage emission stock is ${\tilde{S}}_{k}>{\tilde{S}}_{1}$.

## 4. Conclusions and Prospects

### 4.1. Conclusions

This paper proposes a social welfare maximization game model to analyze the dominant strategy equilibrium of enterprise-1 and enterprise-2 based on the welfare maximization under the total sewage emission control policy.

First, according to the static game equilibrium of complete information, the government implements the total sewage emission control policy for enterprise-1 and enterprise-2. Based on the maximization of enterprise interests, the output and the amount of sewage emission from enterprise-1 and enterprise-2 are obtained under the equilibrium situation. We found that: (1) Under the total sewage emission control policy, a stricter total sewage emission control of an enterprise corresponds to a lower enterprise output of the enterprise and higher outputs of competing enterprises; that is, the profit transfer effect occurs. Specifically, if the total sewage emission control of enterprise-2 is stricter, then the output level of enterprise-2 is lower, and the output level of enterprise-1 is relatively higher. At this time, enterprise-1 gains more profits than enterprise-2 and vice versa. (2) When the government implements a relatively strict total sewage emission control policy for the enterprise, it is beneficial to reduce the amount of sewage emission from an enterprise $\;(E_{i},i=1,\;2)$ but it should have no impact on the amount of sewage emission from a competing enterprise; however, the amount of sewage reduction of both enterprises $\;(\alpha_{i},i=1,\;2)$ increases. Specifically, if the total sewage emission control of enterprise-1 is stricter, then the amount of sewage emission from enterprise-1 will be reduced, but the amount of sewage emission from enterprise-2 will be unchanged; however, the amount of sewage reduction from enterprise-1 and enterprise-2 will increase. The same result will be obtained if the total sewage emission control level of enterprise-2 is stricter. (3) If the government does not provide capital and technical support to enterprise-2, then enterprise-1 and enterprise-2 should implement total quantity control policies with different degrees of rigor to avoid the one-size-fits-all phenomenon. Specifically, existing environmental regulations are implemented at the national level. However, the government should formulate different total sewage emission control policies in line with the normal development of an enterprise according to the scale, benefits, and technical conditions of the enterprise to maximize the social welfare. In this paper, the government is a fair and just decision maker. If the government is corrupt or subject to other circumstances, it will not be able to achieve the goal of maximizing social welfare.

Second, by using the Hamilton–Jacobi–Bellman equation to solve the Markov-perfect Nash equilibrium, when the sewage emission stock in the water resources is larger, the total sewage emission control policy should be stricter. When the sewage emission stock in the water resources is smaller, the total sewage emission control policy should be less strict. Therefore, the government should adjust the total sewage emission control policy in time according to the changes in the sewage stock.

Finally, by analyzing the social welfare effect and comparing sewage stocks, we found that under the total sewage emission control policy, for a given quantity of sewage stock *S*, reducing the sewage emission reduction cost of enterprise-2 is conducive not only to increasing regional welfare but also to reducing the regional sewage stock. Therefore, the government should focus more on enterprises with insufficient capital and poor technical skills and provide financial and technical support, which is more conducive to the maximization of the social welfare in the region.

### 4.2. Prospects

Sustainable development of water resources is a serious and complicated problem. This paper mainly studies the game problem of water pollution, which can provide a decision-making basis for sustainable development of regional water resources. However, this paper still has the following deficiencies.

From water resource allocation to water pollution control, sustainable water resource development is a complex systematic and scientific problem. The social welfare maximization model constructed in this paper only analyses the problem of water pollution control and fails to comprehensively consider water resource distribution and water pollution control; thus, analyzing changes in enterprise benefits more specifically is difficult.

Sustainable water resource development requires business and government efforts. This article assumes that the government completes 20% of the sewage disposal task because the government must complete performance appraisals. At the same time, the government also faces sewage governance pressure, which is difficult; therefore, the government’s required contribution to the sewage-disposal task according to sensitivity analyzes will be examined in future research.

## Figures and Tables

**Figure A1:**
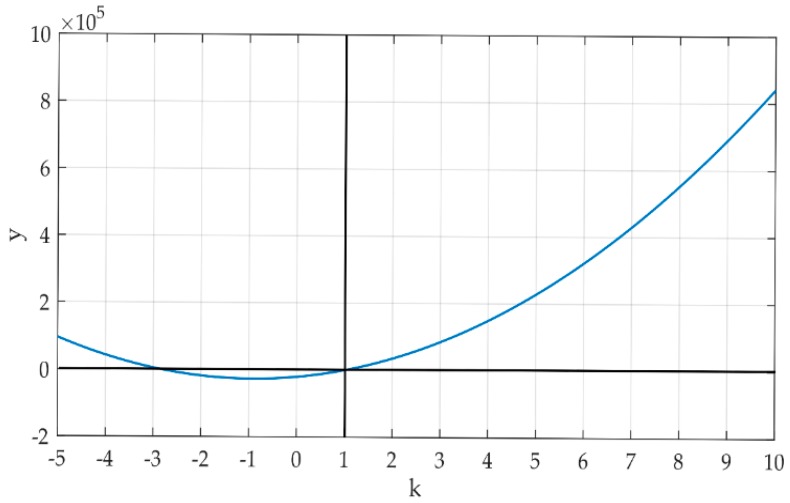
The function image of $\;y=7300k^{2}+13264k-20564$.

**Figure A2:**
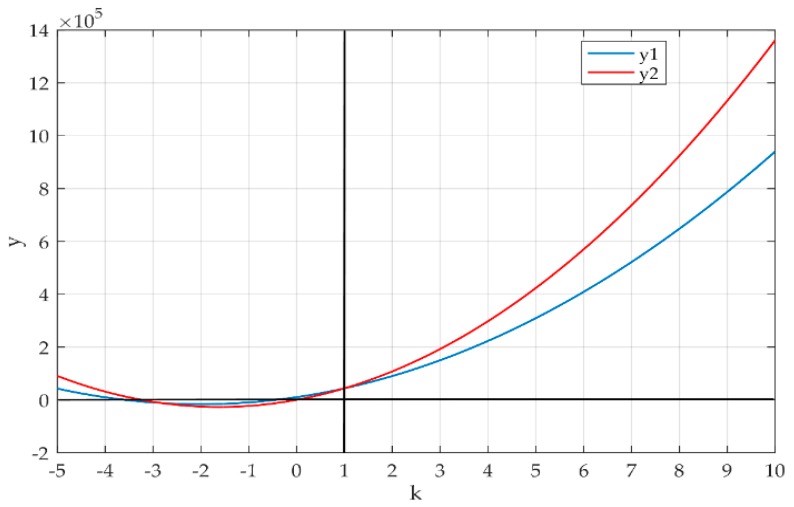
The function images of $y_{1}=6625k^{2}+26659k+10282$ and $y_{2}=10275k^{2}+33291k$.

## Appendix A

**Table A1:** List of symbols.

Symbols	Explanation
$\alpha_{i}$	The amount of sewage reduction from enterprise *i*
β	The amount of sewage treatment from the government
r	Discount rate
δ	The natural degradation rate of sewage
$x_{i}$	The number of goods produced by enterprise *i*
$x_{j}$	The number of goods produced by enterprise *j*
*a*	Constant
*p*	The price of commodities
ε	The enterprise output per unit of the product accompanied by the sewage emission per unit of ε
$\theta_{i}$	Total sewage emission control set by the government for enterprise *i*
$k_{i}$	Sewage emission reduction coefficient of enterprise *i*
λ	Constant
μ	Unit sewage emission fee
ζ	The government’s sewage emission reduction coefficient reflects the government’s sewage treatment intensity
$\pi_{i}$	Profit of enterprise *i*
ρ	Tax rate
$R_{i}$	Revenue of enterprise *i*
*L*	Lagrange function
*S*	Sewage stock
$E_{i}$	The amount of sewage emission from enterprise *i*
*G*	Government revenue
*W*	Welfare function
*D(S)*	Environmental damage function
*V(S)*	Value function
*E(S)*	Sewage emission reduction function

## Appendix B

Proof of $\;E_{k}\left(0\right)>E_{1}\left(0\right)>0$:

(A1) $$\begin{array}{cc} E_{k}\left(0\right)-E_{1}\left(0\right) & =0.8\times \left[\frac{125ak^{2}+405ak+\left(300k^{2}+1236k+776\right)B_{k}}{625k^{2}+2515k+970}-\frac{530a+2312B_{1}}{4110}\right]\\ & =0.8a\left[\frac{25k^{2}+81k+\frac{\left(25k^{2}+81k\right)\rho_{k}}{2\left(r+\delta \right)-\rho_{k}}}{125k^{2}+503k+194}-\frac{53+\frac{53\rho_{1}}{2\left(r+\delta \right)-\rho_{1}}}{411}\right]\\ & =0.8a[\frac{2{\left(r+\delta \right)}^{2}\left(7300k^{2}+13264k-20564\right)}{411\left(125k^{2}+503k+194\right)\left[2\left(r+\delta \right)-\rho_{k}\right]\left[2\left(r+\delta \right)-\rho_{1}\right]}\\ & +\frac{2\left(r+\delta \right)\left[\rho_{k}\left(6625k^{2}+26659k+10282\right)-\rho_{1}\left(10275k^{2}+33291k\right)\right]}{411\left(125k^{2}+503k+194\right)\left[2\left(r+\delta \right)-\rho_{k}\right]\left[2\left(r+\delta \right)-\rho_{1}\right]}] \end{array}$$

where $\rho_{k}=r+2\delta -\sqrt{{\left(r+2\delta \right)}^{2}+16\phi_{2}^{k}}\;\mathrm{and}\;\rho_{1}=\;r+2\delta -\sqrt{{\left(r+2\delta \right)}^{2}+16\phi_{2}^{1}}$.

From this equation, when k > 1, $7300k^{2}+13264k-20564>0$ (as shown in Figure A1) and $0<6625k^{2}+26659k+10282<10275k^{2}+33291k$ (as shown in Figure A2). Because $0>\rho_{k}>\rho_{1}$, $\rho_{k}\left(6625k^{2}+26659k+10282\right)>\rho_{1}\left(10275k^{2}+33291k\right),\;7300k^{2}+13264k-20564>0,$ and $2{\left(r+\delta \right)}^{2}\left(7300k^{2}+13264k-20564\right)+2\left(r+\delta \right)\left[\rho_{k}\left(6625k^{2}+26659k+10282\right)-\rho_{1}\left(10275k^{2}+33291k\right)\right]>0$. Because $2\left(r+\delta \right)-\rho_{k}=r+\sqrt{{\left(r+2\delta \right)}^{2}+16\phi_{2}^{k}}>0$ and $2\left(r+\delta \right)-\rho_{1}=r+\sqrt{{\left(r+2\delta \right)}^{2}+16\phi_{2}^{1}}>0,$ $\;E_{k}\left(0\right)-E_{1}\left(0\right)>0$.

Additionally:

(A2) $$\begin{array}{l} E\left(0\right)=0.8\times \left[\frac{125ak^{2}+405ak+\left(300k^{2}+1236k+776\right)B}{625k^{2}+2515k+970}\right]\\ \;=0.8a\left[\frac{25k^{2}+81k+\frac{\left(25k^{2}+81k\right)\rho }{2\left(r+\delta \right)-\rho }}{125k^{2}+503k+194}\right]\\ \;=0.8a\left[\frac{2\left(r+\delta \right)\left(25k^{2}+81k\right)}{\left(125k^{2}+503k+194\right)\left[2\left(r+\delta \right)-\rho_{k}\right]}\right]>0 \end{array}$$

Thus, $E_{k}\left(0\right)>E_{1}\left(0\right)>0$.
